# Prevalence of intestinal helminth infections in Jiangsu Province, eastern China; a cross-sectional survey conducted in 2015

**DOI:** 10.1186/s12879-019-4264-0

**Published:** 2019-07-10

**Authors:** Yang Dai, Xiangzhen Xu, Jianfeng Liu, Xiaolin Jin, Mingxue Shen, Xiaoting Wang, Jun Cao, Haitao Yang

**Affiliations:** 1 National Health Commission (NHC) Key Laboratory of Parasitic Disease Control and Prevention, Jiangsu Provincial Key Laboratory on Parasite and Vector Control Technology, Jiangsu Institute of Parasitic Diseases, Meiyuan 117, Wuxi City, Jiangsu Province People’s Republic of China; 2 Public Health Research Center, Jiangnan University, Wuxi, People’s Republic of China; 3 Center for Global Health, Nanjing Medical University, Nanjing, People’s Republic of China

**Keywords:** Intestinal parasitic infection, Prevalence, Survey, *Clonorchis sinensis*, Jiangsu province, Eastern China

## Abstract

**Background:**

Intestinal helminth infections are a serious public health problem in developing countries. Jiangsu, an eastern coastal province of China, has an environment conducive to the transmission of intestinal parasites, and suffered human infection rates of 71.75% in 1990. Due to comprehensive anti-transmission measures undertaken throughout the province in the 1990s, the prevalence had decreased to 9.28% in 2002. In order to assess the current epidemic situation for intestinal parasitic infections in Jiangsu province, a province-wide cross-sectional survey was carried out in 2015.

**Methods:**

Surveys were conducted in two main settings; rural (for soil-transmitted parasites) and urban (for *Clonorchis sinensis*), selected through stratified random sampling. Human infection rates were evaluated through the detection of helminth eggs or cysts (oocysts or trophozoites) of intestinal protozoa in fecal samples by microscopy. Secondary intermediate and reservoir hosts were surveyed for *C. sinensis* infection. Questionnaires were completed by each participant to evaluate knowledge, attitude and practice of soil-transmitted parasite and *C. sinensis* avoidance.

**Results:**

115 out of 30153 participants (0.38%) had intestinal helminths or protozoa. There were eight species of helminth detected and the most common parasite was the hookworm *Ancylostoma duodenale*. In rural settings, there were significant differences in infection rates between participants of differing economic status. In urban settings, only four cases of *C. sinensis* infection were detected. However, secondary intermediate and reservoir hosts were found to harbor parasites. The questionnaire survey revealed that 38.42% participants were not aware of how humans become infected by hookworms. Knowledge and awareness of *C. sinensis* was similarly low, with 53.22% participants combining the use of chopping boards for raw and cooked food items when preparing meals.

**Conclusions:**

The prevalence of intestinal parasitic infections in Jiangsu Province in eastern China has decreased from 71.57% in 1990 to 0.38% in 2015. Control measures should now focus on parasitic infections in the elderly and in children, health promotion and the development of alternative detection methods.

**Electronic supplementary material:**

The online version of this article (10.1186/s12879-019-4264-0) contains supplementary material, which is available to authorized users.

## Background

Infection with intestinal parasites, including soil-transmitted helminths (STHs), intestinal protozoa, and food-borne parasites (which can be diagnosed through the detection of eggs/cysts in human stool samples), occurs throughout the world and represents a widespread and serious public health problem in developing countries [1]. The most common intestinal parasites within the group of helminths are STHs, including *Ascaris lumbricoides* (roundworm), *Necator americanus* and *Ancylostoma duodenale* (hookworm), and *Trichuris trichiura* (whipworm), which infect more than 1 billion people, with up to 5.3 billion people at risk of infection with at least one species [2–4]. In China, the overall prevalence of STH infections in 2010 was 11.4%, with 6.8% of these infections caused by *Ascaris lumbricoides* [5]. Food-borne parasites are also an important cause of intestinal parasite infections, and liver flukes are the predominant food-borne parasitic human pathogen. There are an estimated 15.3 million cases of *Clonorchis sinensis*(*C. sinensis*)infection worldwide, and 80% of these infections are concentrated in China [6, 7].

Intestinal parasite infections are important neglected tropical diseases. In general, infection with intestinal parasites causes diarrhea, iron deficiency anemia, growth retardation in children, and other physical and mental health problems [8]. Furthermore, current studies have shown that chronic intestinal parasite infection can affect the spread, severity, and outcome of other infectious diseases, such as tuberculosis, malaria, and viral infections [9–11]. However, *C. sinensis* infection is primarily related to liver and biliary disorders, especially cholangiocarcinoma [12, 13].

Several factors contribute to the high prevalence of intestinal parasites in tropical and sub-tropical countries, including climatic conditions, poor sanitation, and a lack of safe water and adequate toilet facilities [14]. Furthermore, certain behaviors can lead to infection with specific pathogens; for instance, eating raw food can lead to infection with food-borne parasites, and barefooted agricultural work can lead to hookworm infection [13, 15]. Strategies such as anthelmintic drug therapy and water, sanitation, and hygiene improvements can play a significant role in controlling intestinal parasite infections [16, 17]. Also, providing health education and promoting positive health behaviors can help to decrease the incidence of intestinal parasitic infections [14, 18].

Jiangsu is an eastern coastal province of the People’s Republic of China, and has the highest gross domestic product per capita of all of the Chinese provinces. The average temperature is − 1–4 °C in January and 26–29 °C in July, and the annual average rainfall is 800–1200 mm. Its environment is favorable for the transmission of intestinal parasites. The prevalence of intestinal parasite infections was as high as 71.75% in 1990, at which time 18 species of human parasites were present, with 51.5% of residents exhibiting infection with more than one parasite. The implementation of comprehensive control measures throughout the province, including mass drug administration and renovation of the water supply and sewage systems, resulted in a dramatic decrease in the prevalence of intestinal parasites over a 10-year period, to 9.28% in 2002 and nine species of human parasites were detected [6]. In order to assess the current state of intestinal parasite infections in Jiangsu Province, we carried out a province-wide cross-sectional survey in 2015.

## Methods

### Survey design, participants, and organization

Two types of survey settings were selected: rural sites (for soil-transmitted parasites) and urban sites (for *C. sinensis*). A stratified random sampling method was used to select rural sites in Jiangsu Province, according to geographical features, income levels, and the epidemic characteristics of intestinal parasite diseases. A random sampling method was used to select urban sites in Jiangsu Province, according to local dietary habits, human-infection prevalence in 2002, and the epidemic characteristics of clonorchiasis.

One village or one community in a county (city or district) was randomly selected as a survey site, and at least 250 permanent residents (who had lived in the village or community for more than six months) were investigated according to the National Survey Programme of Important Human Parasitic Disease in China. Male and female participants in different age groups and with different occupations were included. More than 85% of the residents were surveyed at each study site.

Jiangsu Institute of Parasitic Diseases was responsible for supervision, data review, and quality control. Staff members from the parasitic diseases department of each municipal- and county-level Center for Disease Control were responsible for the study organization, sample collection, questionnaire administration, field-based testing, and initial data input.

### Types of parasite infection investigated

Human infections in each survey site were identified through the screening of fecal samples for the eggs of *Ascaris lumbricoides*, *Necator americanus*, *Ancylostoma duodenale*, *Trichuris trichiura*, *Enterobius vermicularis*, *Taenia solium, Taenia saginata*, *Clonorchis sinensis*, *Paragonimus westermani*, and other intestinal helminths. At the rural sites, cysts, oocysts, or trophozoites from *Giardia lamblia, Cryptosporidium, Entamoeba histolytica, Entamoeba coli, Balantidium coli*, and other intestinal protozoa were also identified. At urban sites, the prevalence of *C. sinensis* infection in animals, including secondary intermediate hosts (freshwater fish) and reservoir hosts (cats, dogs and pigs), was also investigated.

### Specimen collection, detection, and registration

About 50 g of feces were collected from each participant. The Kato-Katz method was used to detect helminth eggs by microscopy, and the number of eggs and type of species were determined. Two slides were counted for each sample, and the number of eggs per gram was calculated by multiplying the average number of eggs per slide by 24. Hookworm species (*N. americanus* or *A. duodenale*) were identified by the filter paper culture method. Cultures were examined under low power magnification (100 ×) for emerging larvae each day starting on the third day to rule out the presence of filariform larvae. Species were identified by morphological characteristics [19]. Intestinal protozoa were detected by Lugol’s Iodine Stain methodology using a microscope with an ocular micrometer. Species were identified according to the morphological characteristics and size of each cyst (oocysts) [20]. For participants younger than six, a transparent tape test was used to detect pinworm (*E. vermicularis*) eggs around the anus [21].

To detect *C. sinensis* infection among animals, at least 100 wild freshwater fish were collected from natural bodies of water (ponds, rivers, or streams) at each urban site, and the direct compression method was used to detect metacercaria in fish muscle tissue [22]. Furthermore, fecal samples were collected from 20 of each type of animal investigated (including cats, dogs and pigs) at each site, and the Kato-Katz method was used to detect *C. sinensis* eggs.

General information was recorded for each survey site, including geographical features (terrain, altitude, latitude, and longitude), population, and dietary habits. Also, general information for each participant was recorded, including name, gender, date of birth, nationality, occupation, education, and the presence or absence of intestinal parasites.

### Questionnaires

Two different questionnaires were used in this study. Questionnaires were collected from legal guardians if participants were under six years old or from themselves in written format if participants were older than six years. Illiterate participants were informed of the content of questionnaires which were filled out by investigators on their behalf. One questionnaire was used for participants from rural sites and covered basic knowledge (knowledge regarding soil-transmitted parasites, infection routes, hazards, and prevention), attitudes (towards deworming treatments and the changing of habits that could lead to infection), and practices that could lead to becoming infected with soil-transmitted parasitic diseases (not washing hands before eating or after using the toilet, drinking non-boiled water, fertilizing soil with fresh excrement, and working in the fields barefoot). The other questionnaire was used for participants from urban sites, and covered basic knowledge (awareness of *C. sinensis*, hazards, infection routes, and prevention), attitudes (towards eating raw fish, seeking deworming treatment, and the changing of habits that could lead to infection), and practices that could lead to clonorchiasis (using the same chopping board for raw and cooked food, and eating raw freshwater fish/shrimp).

### Data analysis

Epi Info software (version 6) was used to create the database. Double entry and recheck steps were used to ensure data accuracy. Pearson χ^2^ and Fisher’s exact tests were used to investigate associations among qualitative categorical variables using SPSS software (Version 19.0, SPSS Inc., Chicago, Illinois), and a *p*-value of less than 0.05 was considered to be statistically significant. The Bonferroni correction was used for each pairwise comparison.

## Results

### Sampling results

There are 13 prefecture-level cities in Jiangsu Province, which are divided into southern (Nanjing, Wuxi, Changzhou, Suzhou, and Zhenjiang), middle (Nantong, Yangzhou, and Taizhou), and northern areas (Xuzhou, Lianyungang, Huaian, Yancheng, and Suqian), according to geographic locations and levels of social economic development. A total of 117 survey sites were sampled, comprising 69 rural sites and 48 urban sites covering each prefecture-level city and a wide range of different areas and different income levels (Table 1). The raw datasets were shown in Additional file 6.

**Table 1:** Human infection rates in each prefecture-level city in Jiangsu Province

Cities	No. of rural sites	No. of urban sites	No. of positive cases/participants	Positive rate (%)	χ^2^, *P* value
Nanjing	4	0	0/1000	0.00	137.253, < 0.001
Wuxi	4	0	4/1080	0.37	
Changzhou	3	0	2/759	0.26	
Suzhou	4	4	12/2155	0.56	
Zhenjiang	6	8	4/3565	0.11	
Nantong	6	4	3/2509	0.12	
Yangzhou	7	0	6/1780	0.34	
Taizhou	4	8	14/3143	0.45	
Xuzhou	6	10	15/4173	0.36	
Lianyungang	4	4	13/2043	0.64	
Huaian	8	6	10/3601	0.28	
Yancheng	9	4	7/3309	0.21	
Suqian	4	0	25/1036	2.41	
Total:	69	48	115/30153	0.38	–

**Table 2:** Prevalence of human intestinal parasite infection according to demographic and socioeconomic factors

Items	No. of positive cases/participants	Positive rate (%)	χ^2^, P value
Sex:			
Male	43/14,007	0.31	3.811, 0.051
Female	72/16,146	0.45	
Age:			
1–10	9/2890	0.31	10.871, 0.092
11–20	2/1372	0.15	
21–30	6/2496	0.24	
31–40	7/2723	0.26	
41–50	17/5385	0.32	
51–60	25/6016	0.42	
61 or above	49/9271	0.53	
Education:			
No education	31/5430	0.57	13.749, 0.007
Primary school education	43/9884	0.44	
Junior school education	28/9900	0.28	
High school education	13/3786	0.34	
College or above	0/1153	0.00^a^	
Occupation:			
Preschooler	5/1506	0.33	12.542, 0.084
Student	5/2598	0.19	
Worker	8/4313	0.19	
Farmer	81/16,847	0.48	
Public servant	5/1808	0.28	
Retiree	7/1864	0.38	
Unemployed	4/1024	0.39	
Other	0/193	0.00	

^a^ Statistically significant differences (*P* < 0.005), compared to positive rate with no education;

### Total infection rates and parasites involved in human infection

Out of 30,153 participants, 115 (0.38%) were positive for intestinal helminths or protozoan infections. There was no significant difference in the rate of infection between participants from rural sites and those from urban sites (0.42 and 0.33%, respectively; χ^2^ = 1.467, *P* = 0.226). As shown in Table 2, there were also no significant differences between genders, ages, or occupations (χ^2^ = 3.811, *P* = 0.051; χ^2^ = 10.871, *P* = 0.092; χ^2^ = 12.542, *P* = 0.084; respectively). Overall, 99.35% of participants (29,957/30,153) were of Han descent, and there was no significant difference in infection rate between Han participants and participants from minority nationalities (data not shown). However, there was a significant difference in infection rate depending on level of education (χ^2^ = 13.749, *P* = 0.007), with participants educated to college level or above having significantly lower rates of infection compared to participants with no education (χ^2^ = 6.614, *P* = 0.004).

Parasite eggs were detected in fecal samples from 113 participants. The majority of positive cases had mild infections, and only four cases had moderate infections (according to World Health Organization criteria [23]), including two cases of roundworm infection and two cases of hookworm infection. Protozoan cysts (*B. homonis*) were identified in one fecal sample using Lugol’s Iodine Stain method. In addition, pinworm eggs (*E. vermicularis*) were detected in one out of 634 participants under six years old using the transparent tape test. Furthermore, three of the 113 individuals who tested positive for helminth eggs were also infected with a second parasite (roundworm and pinworm, roundworm and whipworm, hookworm and *F. buski*).

**Fig. 1:**
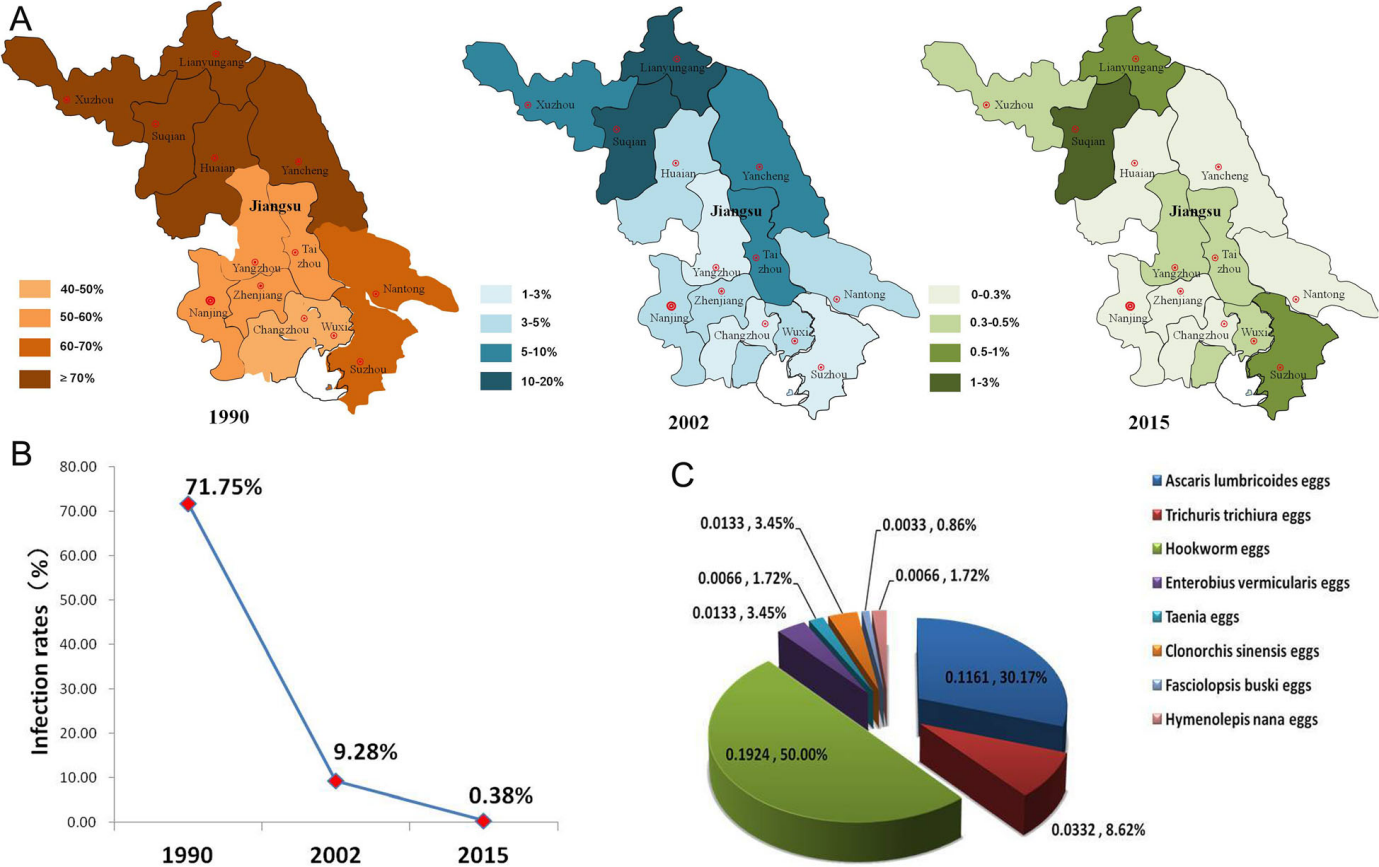
Human intestinal parasitic infection in different areas and at different times in Jiangsu, China. **a** Human infection rates in different areas and at different times; **b**) Total human infection rates through time; **c**) Helminth species distribution in humans in 2015

The parasites identified in human infections in this study are shown in Fig. 1c. Eight species of helminth were detected, and roundworm, hookworm, pinworm, and whipworm accounted for 92.11% of the species found. The most common parasite found was hookworm (58 cases, 50.00%), and *A. duodenale* was the specific species responsible for all of the hookworm infections identified in this study. There were also 35 cases (30.17%) of roundworm infection, which included 25 cases that exhibited fertilized eggs, eight cases that exhibited unfertilized eggs, and two cases that exhibited both fertilized and unfertilized eggs. Furthermore, as shown in Additional file 1: Figure S1, the parasite spectrum detected was different in different areas. There were four, three and eight species of helminth detected in Southern, Middle and Northern Jiangsu, respectively.

### Human infection rates in different prefectural-level cities

The rates of human infection in each prefecture-level city ranged from 0 to 2.41% (Table 1). There was a significant difference in infection rate among different cities (χ^2^ = 137.253, *P* < 0.001). The highest infection rate was in Suqian (2.41%), whilst no positive cases were detected in Nanjing. Table 3 shows the prevalence of human infection from different areas, including southern, middle, and northern Jiangsu. There was a significant difference in the rate of infection in different areas (χ^2^ = 9.246, *P* = 0.01). Furthermore, the rate of infection was significantly higher in northern Jiangsu compared with southern Jiangsu (χ^2^ = 7.446, *P* = 0.006).

**Table 3:** Prevalence of intestinal parasite diseases in different areas of Jiangsu Province

Areas	No. of positive cases/participants	Positive rate (%)	χ^2^, P value
Southern Jiangsu	22/8559	0.26^a^	9.246, 0.01
Middle Jiangsu	23/7432	0.31^b^	
Northern Jiangsu	70/14,162	0.49	

^a^ Statistically significant differences (*P* < 0.01), compared to positive rate from Northern Jiangsu;

^b^ Statistically significant differences (*P* < 0.05), compared to positive rate from Northern Jiangsu;

To date, a cross-sectional survey of intestinal parasitic diseases has been carried out approximately every ten years in Jiangsu Province. Figure 1a and b show the rate of human infection in different areas over time. There has been a significant change the infection rate over time (χ^2^ = 41216.14, *P* < 0.001), and the total rate detected in the current study was significantly lower than that detected in 1990 and 2002 (χ^2^ = 30247.67, P < 0.001; χ^2^ = 2596.27, P < 0.001; Additional file 2: Table S1).

### Parasite infection rate at rural sites

A total of 17,730 participants from 69 rural sites were included in this study, and the total infection rate was 0.42% (Additional file 3: Table S2). There were no significant differences in the infection rates of participants with different ages and levels of education (χ^2^ = 10.340, *P* = 0.107; χ^2^ = 8.447, *P* = 0.066). However, female participants were significantly more likely to be infected than male participants (χ^2^ = 5.201, *P* = 0.023). Table 4 shows the infection rates of participants according to income levels; there was a significant difference in the rate of infection of participants with different income levels (χ^2^ = 26.735, *P* < 0.001). Furthermore, participants with low income levels were significantly more likely to be infected than those with high and middle income levels (χ^2^ = 16.560, P < 0.001; χ^2^ = 16.742, P < 0.001).

**Table 4:** Infection rates of participants with different income levels in rural areas

Income level	No. of positive cases/participants	Positive rate (%)	χ^2^, *P* value
High	20/7277	0.27^a^	26.735, < 0.001
Middle	10/4830	0.21^b^	
Low	44/5623	0.78	

^a^ Statistically significant differences (P < 0.001), compared to positive rate with low income level;

^b^ Statistically significant differences (*P* < 0.001), compared to positive rate with low income level;

### Parasite infection rate at urban sites

A total of 12,423 participants from 48 urban sites were investigated in this study, and the total infection rate was 0.33% (Additional file 3: Table S2). There were no significant differences in infection rates of participants with different genders, ages, and levels of education (χ^2^ = 0.053, *P* = 0.818; χ^2^ = 6.055, *P* = 0.408; χ^2^ = 8.126, *P* = 0.074). Only four cases of *C. sinensis* infection were detected. However, infection with *C. sinensis* was still evident in secondary intermediate and reservoir hosts (Table 5). A total of 5,104 wild freshwater fish (about 30 species) were investigated, and fish from four species were found to be infected with *C. sinensis*. There was a significant difference in the infection rate between species (χ^2^ = 142.874, *P* < 0.001), and *Pseudorasbora parva* showed the highest level of metacercaria in the muscle tissue. No metacercaria were detected in other types of fish, such as crucian carp, grass carp, and silver carp. In total, 1,171 individual animals representing three types of reservoir host (cats, dogs, and pigs) were tested, and *C. sinensis* was detected in individuals from all three species. There was a significant difference in the rate of infection in the different animal types (χ^2^ = 25.337, P < 0.001), and cats were significantly more likely to be infected than dogs or pigs (χ^2^ = 21.200, P < 0.001; χ^2^ = 7.724, *P* = 0.005).

**Table 5:** Prevalence of *Clonorchis sinensis* in second intermediate and reservoir hosts

Animals	No. of positive cases/total cases	Positive rate (%)	χ^2^, P value
Second intermediate host (freshwater fish):			
*Pseudorasbora parva*	104/1752	5.94	142.874, < 0.001
*Abbottina rivularis*	17/1236	1.38	
*Rhodeus ocellatus*	7/176	3.98	
*Hemiculter leucisculus*	4/677	0.59	
Others	0/1263	0.00	
Total:	132/5104	2.59	–
Reservoir host:			
Cats	28/435	6.44^a,b^	25.337, < 0.001
Dogs	3/440	0.68	
Pigs	6/296	2.03	
Total:	37/1171	3.16	–

^a^ Statistically significant differences (P < 0.001), compared to positive rate from dogs;

^b^ Statistically significant differences (P = 0.005), compared to positive rate from pigs;

### Analysis of questionnaire responses

A total of 16,592 (93.58%) valid questionnaires were collected from rural sites. More than 85% of the participants had some knowledge of STHs (were aware of STHs, infection routes, hazards, and prevention) (Additional file 4: Table S3); however, 38.42% of the participants did not know how hookworms infect humans. More than 90% of participants practiced healthy behaviors in their daily life, including washing hands before eating or after using the toilet, not drinking non-boiled water, and not using fresh feces for fertilizer. In addition, 26.61% of the participants worked barefoot in the fields. More than 90% of the participants had a positive attitude toward deworming and changing their habits.

A total of 11,395 (91.73%) valid questionnaires were recovered from urban sites. Knowledge about *C. sinensis* was relatively low (less than 70%) in this population (Additional file 5: Table S4). In total, 87.91% of the participants did not typically eat raw freshwater fish or shrimp; however, 53.22% of participants used the same cutting board for raw and cooked food. Furthermore, more than 95% of participants had a positive attitude toward preventing and treating *C. sinensis* infections.

## Discussion

We carried out a cross-sectional survey of intestinal parasitic infections in Jiangsu Province in eastern China. We found that the human infection rate has decreased from 9.28 to 0.38% over the past 10 years. These data provide a better understanding of the current prevalence and characteristics of intestinal parasitic infections in this region, which can be used to inform the future implementation of control measures.

Three cross-sectional surveys carried out approximately every ten years, show that the rate of human infection has decreased dramatically over the last 30 years [6]. Comprehensive measures including mass drug administration, renovation of the water supply and sewage systems, and health education were implemented in this province in the 1990s. As of 2015, approximately 115.96 million deworming treatments and prophylactic chemotherapy regimens (albendazole and mebendazole) have been administered throughout Jiangsu Province (Data not published). Furthermore, the government has continuously promoted the renovation of the water supply and sewage systems, especially in rural areas. In addition, a public sewage system was in place in 87.52% of rural areas in Jiangsu Province by the end of 2015 (Data not published). Data from this study indicates that the implementation of these measures has had a marked effect on intestinal parasite control.

Previous studies have shown that factors such as gender, age, education, and occupation, may influence the rate of intestinal parasite infections. Our data show that female participants in rural areas had a higher rate of infection than men; this may be because they are more likely to engage in agricultural work and come into contact with soil [19]. We also observed a negative correlation between education, socioeconomic level, and infection rates. This may be explained by healthier habits or better living conditions for participants who had high education levels or were from wealthy areas [24, 25]. We did not observe any significant differences in infection rate between participants based on age and occupation.

The mass migration of rural laborers into urban areas means that the population of rural areas of Jiangsu is primarily comprised of children and the elderly; these groups are at high risk for infection with intestinal parasites, especially STHs. Our data show that elderly individuals are more likely to be infected than individuals from other age groups. The most common parasite identified in this study was the hookworm *A. duodenale*. It is reasonable to presume that this is due to the relatively low awareness of hookworm infection routes and the relatively common practice of peforming agricultural work barefoot, as reported in the questionnaire responses.

Clonorchiasis is endemic in southern (Guangdong and Guangxi Province) and northeastern (Heilongjiang and Jilin Province) China, due to frequent consumption of raw fish and shrimp [12, 13]. Clonorchiasis is also endemic in Jiangsu Province, although at low rates (< 0.1% historically), especially in northern parts of the province. Our data confirmed this low rate of infection (only four positive cases were detected); however, other stages of the life cycle of *C. sinensis* were still found in animals.

Unfortunately, a relatively low level of knowledge about *C. sinensis* and a high rate of risky behaviors (using the same chopping board for raw and cooked food) were observed in this study. Furthermore, dietary habits including eating raw/uncooked freshwater fish or shrimp were prevalent among participants. Therefore, there is a potentially significant risk of human infection in this province, and further health education and food safety risk monitoring should be carried out in the near future.

Traditional pathogen-detecting methods are useful for field studies of parasitic infections as they are low-cost and simple to perform. Several traditional pathogen-detecting methods were used in this study, including the Kato-Katz method for parasite egg detection, Lugol’s Iodine Stain method for protozoa detection, and the direct compression method for detecting metacercaria in fish muscle tissue. The sensitivity of these methods may be lower in the context of low infection rates and mild infections [26, 27]. Thus, some cases may have been missed, even though two slides were prepared from each fecal sample for detection by the Kato-Katz method.

Several molecular methods based on the detection of parasite-specific nucleotide sequences have been developed in recent years, and these methods have satisfactory sensitivity and specificity for detecting parasitic infections [28–30]. Disadvantages such as high cost and the need for a professional operator and specialized equipment, limit their usefulness in the field. An alternative method with high sensitivity and specificity that is simple to perform and low-cost should be developed for parasitic infection surveillance in areas with low levels of infection (such as Jiangsu).

## Conclusion

The prevalence of intestinal parasite infections in Jiangsu Province in eastern China has decreased from 71.57% in 1990 to 0.38% in 2015. Next-step control measures should focus on parasitic infections in children and the elderly, health education (promotion), food safety risk monitoring for food-borne parasites, and the development of alternative detection methods.

## Additional files

Additional file 1: **Figure S1.** The parasite spectrum detected in different areas. A) Southern Jiangsu; B) Middle Jiangsu; C) Northern Jiangsu. (JPG 669 kb)

Additional file 2: **Table S1.** Prevalence of intestinal parasitic diseases in humans over time. (DOCX 14 kb)

Additional file 3: **Table S2.** Prevalence of intestinal parasite infection in rural and urban sites according to different demographic and socioeconomic factors. (DOCX 16 kb)

Additional file 4: **Table S3.** Questionnaire results from participants in rural areas. (DOCX 15 kb)

Additional file 5: **Table S4.** Questionnaire results from participants in town/city areas. (DOCX 15 kb)

Additional file 6: Raw datasets in the present survey. (XLSX 9363 kb)

## Data Availability

All data generated or analysed during this study are included in this published article and its Additional files.
