# Supplementary figures and images for: Prevalence of intestinal helminth infections in Jiangsu Province, eastern China; a cross-sectional survey conducted in 2015
Source: BMC Infect Dis. 2019 Jul 10;19:604. doi: 10.1186/s12879-019-4264-0 (PMC6617619; doi:10.1186/s12879-019-4264-0)

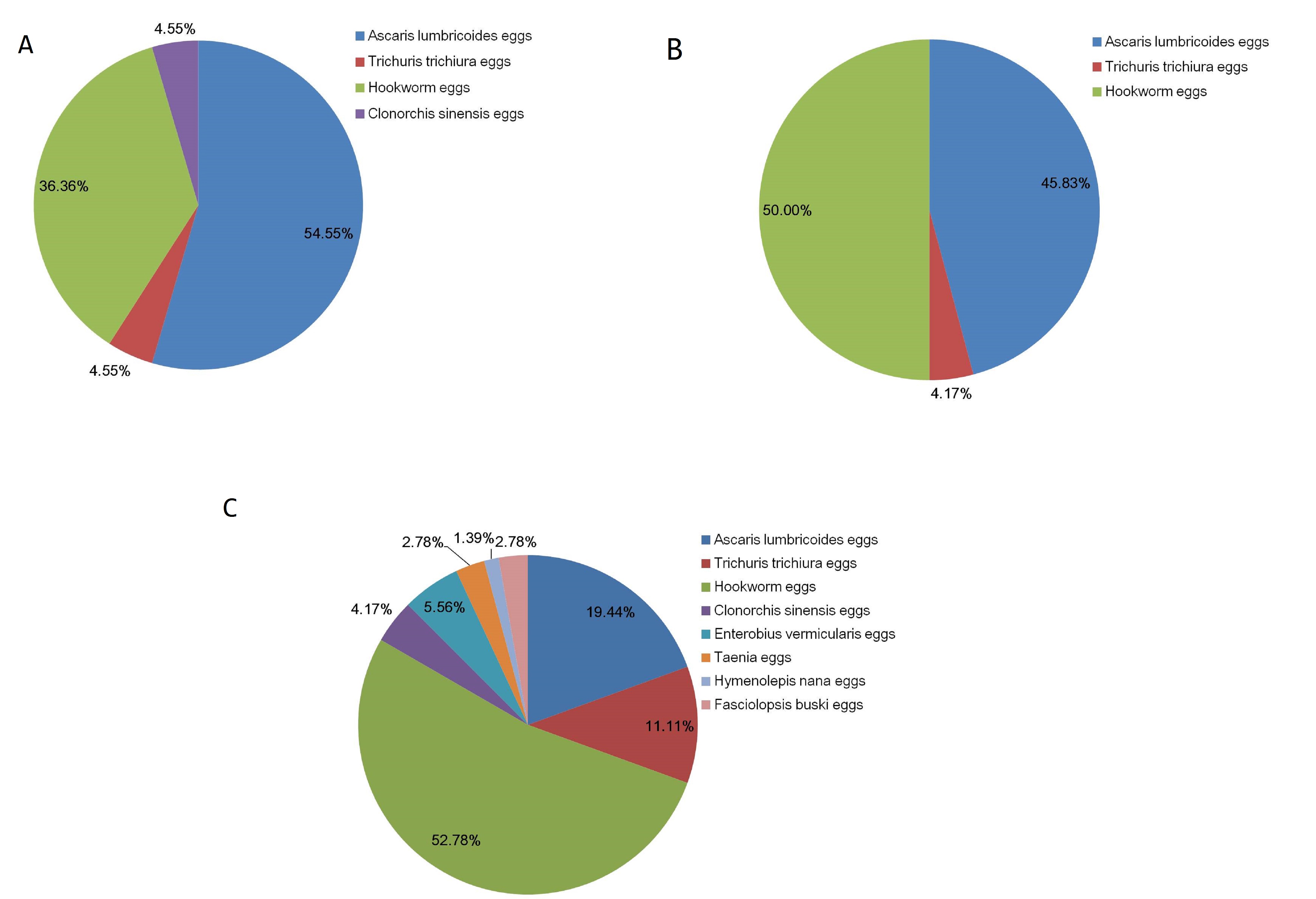

Supplement: Supplementary file 1 — Figure S1. The parasite spectrum detected in different areas. A) Southern Jiangsu; B) Middle Jiangsu; C) Northern Jiangsu. (JPG 669 kb) [file 12879_2019_4264_MOESM1_ESM.jpg]
