# Supplementary material for: Prevalence of intestinal helminth infections in Jiangsu Province, eastern China; a cross-sectional survey conducted in 2015
Source: BMC Infect Dis. 2019 Jul 10;19:604. doi: 10.1186/s12879-019-4264-0 (PMC6617619; doi:10.1186/s12879-019-4264-0)
Supplement: Supplementary file 2 — Table S1. Prevalence of intestinal parasitic diseases in humans over time. (DOCX 14 kb) [file 12879_2019_4264_MOESM2_ESM.docx]

**Additional file 2: Table S1 Prevalence of intestinal parasitic diseases in humans over time**

| **Years** | **No. of positive cases/participants** | **Positive rate (%)** | **χ^2^, P value** |
| --- | --- | --- | --- |
| 1990 | 15,141/21,102 | 71.75 | 41216.14, <0.001 |
| 2002 | 2787/30,031 | 9.28 |  |
| 2015 | 115/30,153 | 0.38^a, b^ |  |

^a^ Statistically significant differences (P < 0.001), compared to total positive rate in 1990;

^b^ Statistically significant differences (P < 0.001), compared to total positive rate in 2002;
