# Supplementary material for: Prevalence of intestinal helminth infections in Jiangsu Province, eastern China; a cross-sectional survey conducted in 2015
Source: BMC Infect Dis. 2019 Jul 10;19:604. doi: 10.1186/s12879-019-4264-0 (PMC6617619; doi:10.1186/s12879-019-4264-0)
Supplement: Supplementary file 3 — Table S2. Prevalence of intestinal parasite infection in rural and urban sites according to different demographic and socioeconomic factors. (DOCX 16 kb) [file 12879_2019_4264_MOESM3_ESM.docx]

**Additional file 3: Table S2** **Prevalence of intestinal parasite infection in rural and urban sites according to different demographic and socioeconomic factors**

| **Items** | **Rural sites** | | | **Urban sites** | | |
| --- | --- | --- | --- | --- | --- | --- |
|  | **No. of positive cases/participants** | **Positive rate (%)** | **χ_2_, P value** | **No. of positive cases/participants** | **Positive rate (%)** | **χ^2^, P value** |
| **Sex:** | | | | | | |
| Male | 25/8331 | 0.30 | 5.201, 0.023 | 18/5676 | 0.32 | 0.053, 0.818 |
| Female | 49/9399 | 0.52 |  | 23/6747 | 0.34 |  |
| **Age:** | | | | | | |
| 1-10 | 3/1679 | 0.18 | 10.340, 0.107 | 6/1211 | 0.50 | 6.055, 0.408 |
| 11-20 | 1/700 | 0.14 |  | 1/672 | 0.15 |  |
| 21-30 | 5/1400 | 0.36 |  | 1/1096 | 0.09 |  |
| 31-40 | 4/1325 | 0.30 |  | 3/1398 | 0.21 |  |
| 41-50 | 9/3281 | 0.27 |  | 8/2104 | 0.38 |  |
| 51-60 | 19/3692 | 0.51 |  | 6/2324 | 0.26 |  |
| 61 or above | 33/5653 | 0.58 |  | 16/3618 | 0.44 |  |
| **Education:** | | | | | | |
| Illiterate | 25/3621 | 0.69 | 8.447, 0.066 | 6/1809 | 0.33 | 8.126,0.074 |
| Primary school education | 24/5952 | 0.40 |  | 19/3932 | 0.48 |  |
| Junior school education | 21/6137 | 0.34 |  | 7/3763 | 0.19 |  |
| High school education | 4/1628 | 0.25 |  | 9/2158 | 0.42 |  |
| College or above | 0/392 | 0.00 |  | 0/761 | 0.00 |  |
| **Total:** | 74/17,730 | 0.42 |  | 41/12,423 | 0.33 |  |
