# Supplementary material for: Prevalence of intestinal helminth infections in Jiangsu Province, eastern China; a cross-sectional survey conducted in 2015
Source: BMC Infect Dis. 2019 Jul 10;19:604. doi: 10.1186/s12879-019-4264-0 (PMC6617619; doi:10.1186/s12879-019-4264-0)
Supplement: Supplementary file 4 — Table S3. Questionnaire results from participants in rural areas. (DOCX 15 kb) [file 12879_2019_4264_MOESM4_ESM.docx]

**Additional file 4: Table S3 Questionnaire results from participants in rural areas**

| **Item** | **Frequency** | **Proportion (%)** |
| --- | --- | --- |
| **Knowledge:** | | |
| 1. Have you ever heard of roundworm, whipworm, or hookworm? | | |
| Yes | 14,121 | 85.11 |
| No | 2471 | 14.89 |
| 2. Do you know how humans become infected with roundworm or whipworm? | | |
| Yes | 13,885 | 83.68 |
| No | 2707 | 16.32 |
| 3. Do you know how humans become infected by hookworm? | | |
| Yes | 10,217 | 61.58 |
| No | 6375 | 38.42 |
| 4. Do you know if infection is harmful to humans? | | |
| Yes | 14,439 | 87.02 |
| No | 2153 | 12.98 |
| 5. Do you know how to prevent infection by roundworm, hookworm, or whipworm? | | |
| Yes | 14,597 | 87.98 |
| No | 1995 | 12.02 |
| **Practice:** | | |
| 7. Do you wash your hands before eating and after using toilet? | | |
| Yes | 15,772 | 95.06 |
| No | 820 | 4.94 |
| 8. Do you drink unboiled water? | | |
| Yes | 1073 | 6.47 |
| No | 15,519 | 93.53 |
| 9. Do you use fresh feces as fertilizer? | | |
| Yes | 1298 | 7.82 |
| No | 15,294 | 92.18 |
| 10. Do you do agricultural work barefoot? | | |
| Yes | 4415 | 26.61 |
| No | 12,177 | 73.39 |
| **Attitude:** | | |
| 11. Would you agree to pay for the cost of deworming drugs? | | |
| Yes | 15,481 | 93.30 |
| No | 1111 | 6.70 |
| 12. Would you agree to change your present habits that could lead to parasite infection? | | |
| Yes | 15,990 | 96.37 |
| No | 602 | 3.63 |
