# Supplementary material for: Prevalence of intestinal helminth infections in Jiangsu Province, eastern China; a cross-sectional survey conducted in 2015
Source: BMC Infect Dis. 2019 Jul 10;19:604. doi: 10.1186/s12879-019-4264-0 (PMC6617619; doi:10.1186/s12879-019-4264-0)
Supplement: Supplementary file 5 — Table S4. Questionnaire results from participants in town/city areas. (DOCX 15 kb) [file 12879_2019_4264_MOESM5_ESM.docx]

**Additional file 5: Table S4 Questionnaire survey results from participants in town/city points**

| **Items** | **Frequencies** | **Proportion (%)** |
| --- | --- | --- |
| **Knowledge:** | |  |
| 1. Have you ever heard clonorchiasis? | | |
| Yes | 7891 | 69.24 |
| No | 3504 | 30.75 |
| 2. Do you know how to infect with *Clonorchis sinensis*? | | |
| Yes | 7279 | 63.88 |
| No | 4116 | 36.12 |
| 3. Do you know the hazard of *Clonorchis sinensis* infection? | | |
| Yes | 6599 | 57.91 |
| No | 4796 | 42.09 |
| 4. Do you know how to prevent infection by *Clonorchis sinensis*? | | |
| Yes | 7712 | 67.68 |
| No | 3683 | 32.32 |
| **Practice:** |  |  |
| 5. Do you use cutting board separately for raw and cooked food in your kitchen? | | |
| Yes | 5331 | 46.78 |
| No | 6064 | 53.22 |
| 6. Do you eat raw or uncooked freshwater fish or shrimps in your normal life? | | |
| Yes | 1378 | 12.09 |
| No | 10017 | 87.91 |
| **Attitude:** |  |  |
| 7. Would you like to try eat raw or uncooked fish if there is a risk for *Clonorchis sinensis* infection? | | |
| Yes | 64 | 0.56 |
| No | 11331 | 99.44 |
| 8. Would you agree to pay for the treatment when you infected with *Clonorchis sinensis*? | | |
| Yes | 11309 | 99.25 |
| No | 86 | 0.75 |
| 9. Would you continue to eat raw or uncooked fish or shrimps when you have cured of clonorchiasis? | | |
| Yes | 378 | 3.32 |
| No | 11017 | 96.68 |
